# Hot electron-driven electrocatalytic hydrogen evolution reaction on metal–semiconductor nanodiode electrodes

**DOI:** 10.1038/s41598-019-42566-3

**Published:** 2019-04-17

**Authors:** Ievgen I. Nedrygailov, Song Yi Moon, Jeong Young Park

**Affiliations:** 1 Center for Nanomaterials and Chemical Reactions, Institute for Basic Science, Daejeon, 305-701 Republic of Korea; 2 Department of Chemistry and Graduate School of EEWS, Korea Advanced Institute of Science and Technology (KAIST), Daejeon, 305-701 Republic of Korea

**Keywords:** Surface spectroscopy, Chemical physics

## Abstract

Hot electrons generated on metal catalysts influence atomic and molecular processes, leading to hot electron-driven catalytic reactions. Here, we show the acceleration of electrocatalytic hydrogen evolution caused by internal injection of hot electrons on Pt/Si metal–semiconductor electrodes. When a forward bias voltage is applied to the Pt/Si contact, hot electrons are injected. The excess energy of these electrons allows them to reach the Pt/electrolyte interface and reduce the adsorbed hydrogen ions to form H_2_ (2H^+^ + 2e^−^→H_2_). We show that the onset potential of the hydrogen evolution reaction shifts positively by 160 mV while the cathodic current exhibits an 8-fold increase in the presence of hot electrons. The effect disappears when the thickness of the Pt film exceeds the mean free path of the hot electrons. The concept of a hot electron-driven reaction can lead to the development of a novel mechanism for controlling reactivity at liquid–solid interfaces.

## Introduction

Constant population growth in areas with limited resources pushes researchers around the globe to search for more and more effective ways of conducting chemical reactions at reduced temperatures and ambient conditions^1^. Thus, there is considerable interest in the development of technology for improved activation of chemical reactions using electric charge carriers. Unlike conventional catalysis where chemical reactions are activated by thermal energy, charge-carrier-driven catalytic reactions proceed through the selective activation of chemical bonds by the energy of highly excited (hot) electrons or holes^2–5^. Undesirable energy losses occurring with thermally activated reactions can be avoided by hot electron-mediated processes. In addition, because of the excess energy carried by the hot charge carriers, chemical reactions with a high activation barrier can occur that are impossible with thermal activation^3,4^.

**Figure 1:**
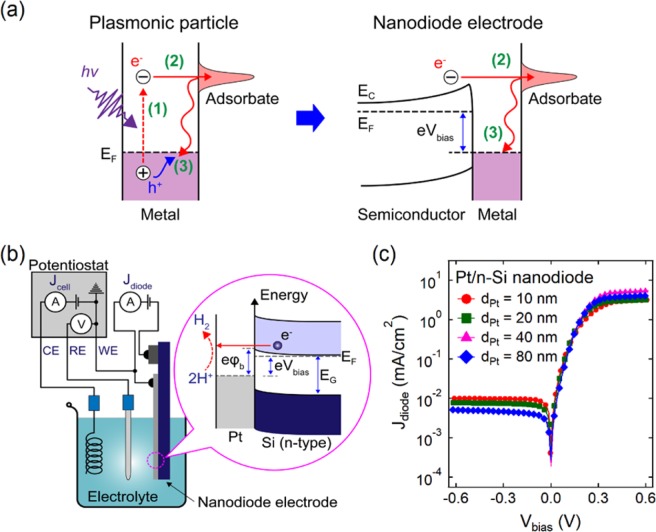
Scheme of the hot electron-driven HER. (**a**) Hot electron injection in a clean plasmonic metal and nanodiode electrode based on a metal–semiconductor contact showing (1) photoexcitation of an electron-hole pair, (2) hot electron injection, and (3) recombination via thermalization of hot carriers. (**b**) Schematic of a three-electrode electrochemical setup with a Pt/Si nanodiode used as the working electrode. Inset: the energy band diagram of the nanodiode electrode. Here, *φ*_*b*_ is the Schottky barrier height, ***E***_***F***_ is the Fermi level, ***E***_***G***_ is the band gap, and ***V***_**bias**_ is the applied bias voltage. (**c**) Current–voltage characteristics of the Pt/Si nanodiode electrodes with varied Pt film thickness. Measurement of the current–voltage curves occurred while the nanodiode electrodes were immersed in 0.5 M H_2_SO_4_.

At present, localized surface plasmon resonance (LSPR) is considered one of the most promising strategies for controlling surface chemistry with hot charge carriers^4–6^. LSPR is the collective oscillation of metal electrons that can be excited by incident light at the interface between a metal nanoparticle and a dielectric (or semiconductor) environment. During decay, LSPR can generate a significant flux of hot electrons and holes with excess energies of several electron volts. In clean metals, plasmon-induced charge carriers can be injected into the adsorbate to drive a photochemical reaction or they disappear as a result of thermalization (Fig. 1a). To reduce the recombination rate of the hot carriers and thereby increase the efficiency of the metal nanoparticles as catalysts, a Schottky contact can be used, which is created by depositing metal nanoparticles on a semiconductor surface^7–11^. Much work has been done studying plasmon-induced reactions of various types ranging from the simplest dissociation of molecules^4,12^ to more complex surface reactions^13–20^. In particular, a number of studies^14,21–25^ have shown a significant improvement in the efficiency of different catalysts for the hydrogen evolution reaction (HER) caused by the generation and transfer of hot electrons. A more detailed review of hot-electron-driven chemistry can be found in a recent paper by Wei and co-workers^7^. However, because of its complexity, the detailed mechanism for energy transfer from a plasmonic nanoparticle to a chemisorbed molecule remains poorly understood^15,26^. The task is further complicated by the fact that catalysts based on LSPR are often multicomponent core-shell or antenna-like nanostructures containing both plasmonic and catalytic materials^15,27^. The properties of such nanostructures can change over the course of a photocatalytic reaction because of the presence of adsorbates^26,28,29^. Therefore, further progress in studying the chemistry of hot-charge carriers requires the development of new research methods that include these factors.

In this report, we present a simple approach that allows us to create a distribution of hot electrons with excess energy on the order of electron volts in Schottky structures similar to those used in plasmonic photocatalysis. The distribution of hot electrons is generated by the injection of charge through a potential barrier formed at a metal–semiconductor (MS) contact (Fig. 1a) that is integrated into the working electrode of a conventional three-electrode electrochemical cell. The features of this approach include the ability to precisely control the generation of hot charge carriers and the possibility for obtaining quantitative information on the intensity of the hot electron flux and energy threshold. The electrodes with built-in MS contacts considered in this paper are composed of planar thin film layers. The generation of hot charges occurs via an external electric field applied to the Pt/Si Schottky contact, not photoexcitation. These electrodes make it possible to investigate the process of hot carrier injection, which is similar to that occurring in plasmonic nanostructures, under very well controlled conditions and thereby provide new opportunities for understanding the basics of this process. Information obtained using electrodes with MS contacts of different configurations can be used in the design of materials for plasmonic photocatalysis, as well as for the development of a methodology for assessing their efficiency. We note that the use of n-type Si substrates as the basis for the electrodes mainly results from the well-studied properties of Pt/Si Schottky contacts, as well as the well-established fabrication process, which allows for the manufacture of electrodes with desired properties. In addition, because of good availability and relatively low cost, Si is often considered a promising material for manufacturing electrodes for hydrogen production^30–34^.

The possibility of using hot electron injection from electrodes with built-in MS contacts to increase the rate of electrochemical reactions was first proposed and justified by Frese *et al*.^35^. The ability to influence the rate of electrochemical reactions on the surface of various electronic devices was also shown in pilot studies^36,37^. It was also recently shown that MS contacts can be used to detect hot electrons from non-adiabatic reactions with a liquid reagent^38^. However, sufficient experimental verification of the proposed concept is still missing. Here, using planar Pt/Si nanodiode electrodes, we study the electrocatalytic hydrogen evolution reaction (HER) driven by hot electrons. We show that hot electron injection leads to a drastic positive shift of the onset potential for the HER. The cathodic current associated with H_2_ production also demonstrates an 8-fold increase. We show that the magnitude of the HER acceleration depends on the flux of hot electrons.

## Results and Discussion

Electrochemical measurements were carried out in a standard three-electrode cell (schematic is shown in Fig. 1b). In this cell, a Pt film supported on an n-Si substrate and a Pt wire are used as the working electrode (WE) and counter electrode (CE), respectively; the n-Si support was also connected to a Ti/Au Ohmic contact. All potentials are measured using an Ag/AgCl reference electrode (RE) and expressed vs. the reversible hydrogen electrode (RHE) scale (see Supporting Information). The Pt film and n-Si support with an Ohmic contact constitutes a thin film Schottky diode, called a Pt/Si nanodiode electrode (NDE). The rectifying properties of the NDE are clearly seen from the current–voltage curves shown in Fig. 1c. The Schottky barrier height of 0.8 eV was determined by fitting the current–voltage curves to the thermionic emission equation (Supporting Information, Fig. S1). An electrical circuit consisting of a DC power supply and a measuring circuit was connected to the Pt film and Ohmic contact on the n-Si support. This circuit, which is completely independent of the electrochemical workstation, was used to supply a bias voltage to the NDE. This experimental setup configuration made it possible to inject hot electrons over the Pt/Si contact by applying a bias voltage of a suitable size and polarity, and to simultaneously study the effect of the injected hot electrons on the HER. The entire NDE, with the exception of the Pt film, was coated with a silicone sealant so that only the Pt surface with an area of 0.8 cm^2^ was in contact with the electrolyte (see Supporting Information).

**Figure 2:**
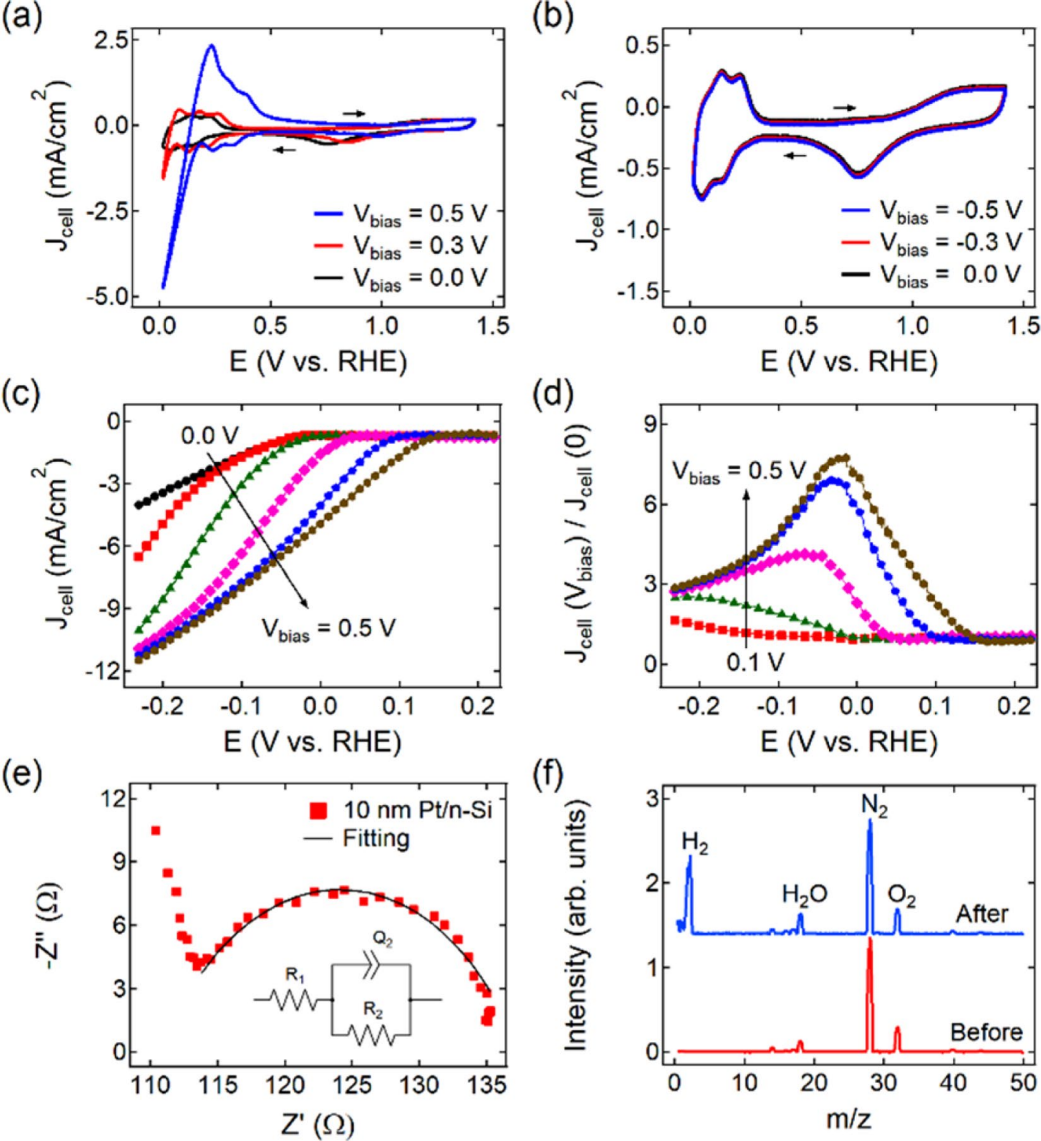
Acceleration of HER on the Pt/Si nanodiode electrodes. (**a**,**b**) Cyclic voltammetry (CV) and (**c**) linear sweep voltammetry (LSV) curves of a 10 nm Pt/Si nanodiode electrode measured at different bias voltages applied between the Pt film and the n-Si substrate (scan rates of 0.1 V·s^−1^ for CV and 1 mV·s^−1^ for LSV). (**d**) Enhancement of the cathodic current of the HER resulting from the nanodiode electrode bias. (**e**) Typical electrochemical impedance spectroscopy (EIS) data for a 10 nm Pt/Si nanodiode electrode. Inset: Equivalent circuit used for fitting the EIS data. (**f**) Typical quadrupole mass spectrometry spectra of the gas evolved from the forward-biased 10 nm Pt/Si nanodiode electrodes. The spectrum of ambient air before the experiment is shown for reference. All measurements are carried out in 0.5 M H_2_SO_4_.

The electrochemically active surface of the Pt/Si NDE was characterized by cyclic voltammetry (CV) measurements. The experiments were run across a potential range of 0 to 1.4 (V vs. RHE) at a scan rate of 0.1 V·s^−1^. Figure 2a shows a typical CV curve measured on the NDE with a 10 nm thick Pt film in the absence of a bias voltage (V_bias_ = 0 V). All the observed features of the CV curve correspond to those reported in the literature^39^. The cathodic current corresponding to the HER (J_cell_) is clearly seen near the zero potential. As shown in Fig. 2a, the CV curve changes dramatically when a forward bias voltage is applied. This bias causes the injection of hot electrons from the n-Si support to the Pt film. A sharp increase in the cathodic current is observed in the hydrogen region of the CV curve. However, applying a reverse bias voltage to the NDE does not lead to any change in the shape of the CV curve (Fig. 2b). To further study the effect caused by forward biasing of the Pt/Si NDEs on the rate of the HER, linear sweep voltammetry (LSV) curves were measured at a scan rate of 1 mV·s^−1^. Figure 2c shows the polarization curves for the 10 nm Pt/Si NDE measured at different forward bias voltages. The LSV curves are corrected for Ohmic losses. The cathodic current clearly exhibits a tendency to increase with larger bias voltages. Thus, with a bias value of V_bias_ = 0.5 V, the cathodic current is up to 8 times greater than that observed in the absence of biasing (Fig. 2d). The effect of the applied bias is fully reversible: turning off the bias results in the cathodic current returning to its initial value. The onset potential of HER (i.e., the potential where the tangents cross at the non-faradaic and faradaic zones of the LSV curve (Supporting Information, Fig. S2)) shows a tendency to shift to more positive values when the NDE is forward-biased. At V_bias_ = 0.5 V the onset potential is approximately ΔE = 160 mV more positive than that for the NDE in a state of equilibrium (V_bias_ = 0 V). Figure 2e shows a typical Nyquist plot and the equivalent circuit, obtained for the 10 nm Pt/Si NDE from electrochemical impedance spectroscopy (EIS) measurements (see Supporting Information). To confirm that the increase in the cathodic current on the forward-biased Pt/Si NDE results from more intense H_2_ production, the gas released from the surface of the NDE was collected and its composition was analyzed using a Hiden Analytical Limited HPR-20 QIC quadrupole mass spectrometer. As shown in Fig. 2f, the increase in the cathodic current of the forward-biased NDE is indeed accompanied by H_2_ evolution.

**Figure 3:**
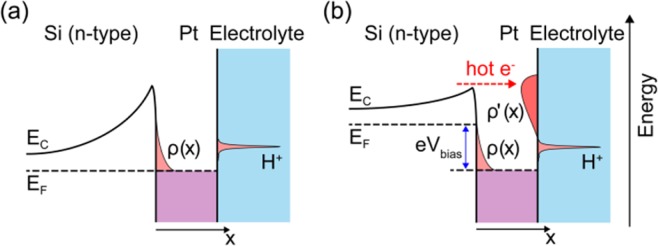
Mechanism for HER enhancement by the injection of hot electrons. Energy band diagram of the Pt/Si nanodiode electrode (**a**) in a state of equilibrium (V_bias_ = 0 V) and (**b**) when a forward bias voltage is applied.

**Figure 4:**
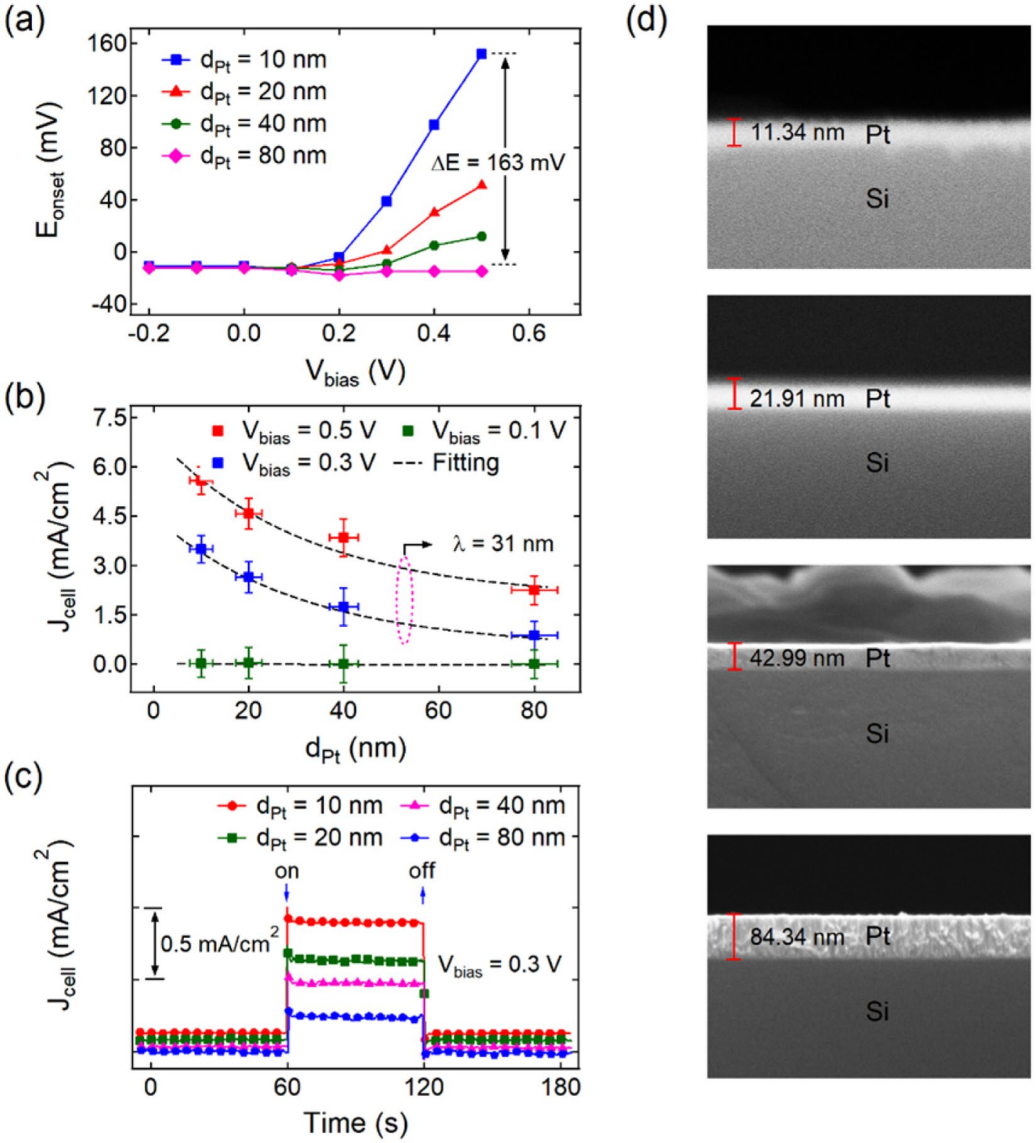
Effect of Pt film thickness on HER enhancement. (**a**) Onset potential of the HER and (**b**) cathodic current as functions of applied bias voltage measured on the Pt/Si nanodiode electrodes with varied Pt film thickness. (**c**) Response of the cathodic current when applying a forward bias voltage to the Pt/Si nanodiode electrode. The measurement of cathodic current for (**b**,**c**) is performed at the potential of the working electrode E = −0.1 (V vs RHE). All measurements were carried out in 0.5 M H_2_SO_4_. (**d**) SEM images of the cleaved cross-sections of the Pt/Si contacts.

To understand the mechanism for accelerating the HER with hot electrons, consider the Pt/Si and Pt/electrolyte interfaces at a state of equilibrium (V_bias_ = 0 V) and when a bias voltage is applied. Let us first look at the energy band diagram for the Pt/Si NDE (Fig. 3a). In the absence of a bias voltage, the net current across the Pt/Si contact is zero because of the Schottky barrier. Free electrons transferred to the Pt film when the MS contact first formed accumulate in a very thin layer close to the Pt/Si interface because of the strong screening effect. This concentration of free electrons rapidly decreases when moving away from the contact and can be approximated by an exponential function: ${\boldsymbol{\rho }}({\boldsymbol{x}})={{\boldsymbol{\rho }}}_{{\bf{m}}{\bf{a}}{\bf{x}}}{\bf{e}}{\bf{x}}{\bf{p}}(-{\boldsymbol{x}}/{{\boldsymbol{l}}}_{{\bf{T}}{\bf{F}}})$^40,41^. The quantity ***l***_**TF**_ is known as the Thomas–Fermi screening length^42^. For platinum, it is estimated to be only 0.5 Å^40,42^ which agrees well with the studies reported by Lykhach *et al*. for platinum/ceria catalysts^41^. Thus, for NDEs where the thickness of the Pt film considerably exceeds ***l***_**TF**_, the activity for the HER is dominated by the properties of the platinum, while the metal/support interactions are largely suppressed by Thomas–Fermi screening. The onset potential for the HER is determined by the energy necessary to transfer an electron from the *d*-band in the Pt film to a hydrogen ion in the electrolyte^43^. When the Pt/Si NDE is forward-biased, the Fermi level in the semiconductor rises relative to the Fermi level in the metal (Fig. 3b). This breaks the equilibrium state and leads to the injection of hot electrons over the Schottky barrier. These hot electrons carry an excess energy equal to or slightly higher than the barrier height and move ballistically – without scattering – through the Pt film. Since the distance that hot electrons can move in a Pt film is on the order of 10 nm^35^, the injected hot electrons can easily reach the Pt/electrolyte interface where they can reduce hydrogen ions to form H_2_ ($2{{\bf{H}}}^{+}+2{{\bf{e}}}^{-}\to {{\bf{H}}}_{2}$). In this case, the electrocatalytic properties of the Pt/Si NDE are determined by the distribution of hot electrons (${\boldsymbol{\rho }}^{\prime} ({\boldsymbol{x}})$) in the Pt film rather than its intrinsic properties. The distribution of hot electrons in turn depends on energy band bending at the MS contact as well as the peculiarities of hot electron transfer across the NDE. Thus, by changing the properties of the MS contact and by varying the applied bias voltage, it is possible to create different distributions of hot charge carriers in the working electrode and to investigate their effect on the reaction rate. This last statement is illustrated in Fig. 4a where the onset potential of the HER is shown to be a function of bias voltage.

To estimate the quantum efficiency (QE) of the HER driven by hot electron injection, the total amount of charge involved in the reaction on the NDE with (Q_bias_) and without (Q_0_) bias voltage was measured as the area under the cathodic current vs. time curve (${\bf{Q}}={{\boldsymbol{\int }}}_{{\bf{0}}}^{{\bf{t}}}{{\boldsymbol{J}}}_{{\bf{c}}{\bf{e}}{\bf{l}}{\bf{l}}}{\bf{d}}{\bf{t}}$). The QE was then calculated according to

1 $${\bf{Q}}{\bf{E}}=\frac{{\rm{\Delta }}{\bf{Q}}}{{{\bf{Q}}}_{{\bf{d}}{\bf{i}}{\bf{o}}{\bf{d}}{\bf{e}}}}\times \mathrm{100}{\boldsymbol{ \% }}$$

where ${\rm{\Delta }}{\bf{Q}}={{\bf{Q}}}_{{\bf{b}}{\bf{i}}{\bf{a}}{\bf{s}}}-{{\bf{Q}}}_{0}$ is the increment of charge involved in the HER after biasing the NDE, and Q_diode_ is the corresponding charge transferred across the NDE. Electrodes with a 10 nm thick Pt film and bias voltages of V_bias_ = 0.1–0.5 V obtained a yield of 5.6–8.5%. It is interesting to compare these data for QE of the Pt/Si NDE with data obtained from plasmonic MS structures. Theoretical calculations performed for the case of plasmon-enhanced internal injection of electrons in MS Schottky contacts with perfect optical absorption indicate a maximum QE of 7–8%^7,44,45^. This agrees well with our estimates for the efficiency of hot electron injection in the NDE. We assume that such a high QE value for the Pt/Si NDE, which is close to the theoretical maximum, may primarily result from the high quality of the Schottky contacts because of the use of high-purity Si substrates and the homogeneity of the Pt films, which reduce the scattering of the injected hot electron flux. The experimentally measured internal QE for the various plasmonic photovoltaic and photocatalytic structures on a Schottky contact, as a rule, do not exceed 3%^7^, which is apparently caused by low-efficiency light absorption and the large concentration of defects that accelerate the recombination of photo-excited carriers.

The distance hot electrons travel in metal films is limited by scattering processes^35,38,46,47^. Thus, the concentration of hot electrons at the Pt/electrolyte interface is proportional to  ${\bf{e}}{\bf{x}}{\bf{p}}({-d}_{{\boldsymbol{P}}{\boldsymbol{t}}}/{\boldsymbol{\lambda }})$^35^, where ***λ*** is the ballistic mean free path. Under these conditions, it is reasonable to assume that the current of the HER should decrease exponentially as the film thickness increases

2 $${{\boldsymbol{J}}}_{{\bf{c}}{\bf{e}}{\bf{l}}{\bf{l}}}={{\boldsymbol{J}}}_{0}{\bf{e}}{\bf{x}}{\bf{p}}(-{{\boldsymbol{d}}}_{{\boldsymbol{Pt}}}/{\boldsymbol{\lambda }})$$

To verify this hypothesis, NDEs with Pt film thicknesses of 10–80 nm were fabricated and tested under identical reaction conditions. Figure 4b shows the cathodic current of the HER measured from the forward-biased NDEs with varying Pt film thicknesses. In accordance with Eq. 2, the current decreases exponentially with increasing Pt film thickness, which is convincing evidence that the observed acceleration of the HER is a result of hot electron injection. Similarly, the positive shift in the onset potential of the HER decreases significantly when the Pt film thickness increases (Fig. 4a). According to the data shown in Fig. 1c, the conductive properties are similar for all the NDEs, regardless of the Pt film thickness. The similar current–voltage curves for the different Pt thicknesses exclude the possibility for increasing the activity of the NDEs with respect to the HER using Joule heating caused by current flowing through the MS contact. The non-thermal mechanism for accelerating the HER is also indicated by the instantaneous response of the cathodic current to biasing (Fig. 4c). Note that the presence of defects and inhomogeneity has a significant impact on the transport of hot charges across the MS structures^35^. To assess the quality of the Pt/Si contacts, we used cross-sectional scanning electron microscopy (Fig. 4d). For all thicknesses of the Pt/Si NDE, the Schottky contact is fairly uniform, which also explains the good reproducibility of the data in the Fig. 1c.

It should be noted that a similar effect of the acceleration of electrocatalytic reactions on different electronic devices was already reported. For example, Diesing *et al*. studied electrochemical reactions with hot electrons on the surface of MIM tunnel structures^36^. Koshida *et al*. recently showed the acceleration of the HER as a result of the external emission of hot electrons using electrodes composed of a Au film deposited onto a layer of nano-crystalline silicone^37^. Mai *et al*. demonstrated the HER tuned on field-effect VSe_2_ and MoS_2_ nanosheet-based devices^48,49^. Here, we demonstrate for the first time an increase in the electrocatalytic activity of metal–semiconductor catalysts from internal transport of hot electrons. The excess energy of hot electrons in the present study is well below the Pt work function, which is approximately 5.65 eV^50,51^. Thus, the possibility of external electron emission from the Pt film into the electrolyte is unlikely. This suggests an intriguing method for studying chemical reactions on the surface of Schottky structures similar to those used in plasmonic photocatalysis with the use of excited charge carriers, which can lead to the development of a novel mechanism for controlling catalytic properties with a non-thermally activated process.

In summary, we demonstrated that the electrocatalytic hydrogen evolution reaction (HER) can be accelerated by injecting hot electrons on the surface of nanodiode electrodes (NDEs). The NDE is composed of a Pt film supported on an n-Si substrate and can serve as the working electrode in a three-electrode electrochemical cell. It can also be used as a source of hot electrons injected towards the Pt/electrolyte interface when a forward bias voltage is applied. We show that the onset potential of the HER shifts positively by 160 mV while the cathodic current exhibits an 8-fold increase in the presence of hot electron injection. The quantum efficiency of this effect is estimated to be on the order of 5.6–8.5%. The magnitude of the acceleration of the HER decreases exponentially as the thickness of the Pt film increases, which excludes the possibility for acceleration of the HER by Joule heating of the Schottky contact caused by current flow. The approach presented here using NDEs can serve as a platform for in-depth studies of the mechanisms for chemical reactions activated by hot charge carriers.

## Methods

### Fabrication and characterization of nanodiode electrodes

To manufacture the Pt/Si nanodiode electrodes, the following sequence was used: First, the n-type Si (100) substrates (*ρ* = 1–10 Ω·cm) were etched in a buffered oxide etch solution, composed of NH_4_F and HF in water, for 150 s at room temperature. Ohmic contacts were then fabricated on the Si surface by e-beam deposition of a 50 nm thick Ti film, followed by the evaporation of a 50 nm thick Au film through a stainless-steel mask. At the final stage, Pt films with a contact area of 1.16 cm^2^ were deposited in a similar manner. The quality of the surface of the Pt films and the Pt/Si contacts was controlled using scanning electron microscopy (Fig. 4d) and atomic force microscopy (see Supporting Information, Fig. S3). Before immersion in the electrolyte, the nanodiode electrode was placed on a holder made of glass and the entire surface of the electrode, except for the platinum surface with an area of 0.8 cm^2^, was coated with a silicone sealant. The contact between the nanodiode electrode and the external electrical circuit was made via clamping contacts made of gold wire fixed on the glass holder.

Current–voltage curves were measured to characterize the Pt/Si nanodiode electrodes (Fig. 1c). According to the thermionic emission theory, the current–voltage curves for a Schottky barrier follow the equation

$${J}_{{\rm{diode}}}={J}_{{\rm{S}}}[\exp (\frac{e({V}_{{\rm{bias}}}-{J}_{{\rm{diode}}}{R}_{{\rm{ser}}})}{n{k}_{{\rm{B}}}T})-1]$$

where ${J}_{{\rm{S}}}={A}^{\ast }{T}^{2}\exp (-\frac{e{\phi }_{b}}{{k}_{{\rm{B}}}T})$ is the saturation current, ${A}^{\ast }$ is the Richardson constant, *T* is the temperature, *e* is the elementary charge, *φ*_*b*_ is the Schottky barrier height, *k*_B_ is the Boltzmann constant, V_bias_ is the voltage, *R*_ser_ is the series resistance, and *n* is the ideality factor. In the forward-biased regime, the parameters describing the properties of the Schottky barrier (*φ*_*b*_, *R*_ser_, and *n*) can be found by fitting the reverse function *V(J)* to the experimentally measured current–voltage curves

$${V}_{{\rm{bias}}}({J}_{{\rm{diode}}})=\frac{n{k}_{{\rm{B}}}T}{e}\,\mathrm{ln}(\frac{{J}_{{\rm{diode}}}}{{J}_{{\rm{S}}}})+{J}_{{\rm{diode}}}{R}_{{\rm{ser}}}$$

Using the procedure described above, the following values were obtained: *φ*_*b*_  = 0.81 ± 0.01 (eV), *R*_se_ = 80 ± 5 (Ω), and *n* = 1.1 ± 0.1. Note that the barrier height agrees well with the predicted value of 0.8 eV based on the electron work function for polycrystalline Pt (5.65 eV) and n-type Si (4.85 eV) obtained using the photoelectric effect and contact potential difference measurements^51^. This value also fits the Schottky barrier height of 0.85 eV measured elsewhere for various designs of Pt/Si diodes^50,52,53^. The ideality factor close to unity indicates good quality and uniformity of the contact between the Si substrate and the Pt film, which was also confirmed using cross-sectional SEM images (Fig. 4d).

### Electrochemical measurements

The electrochemical measurements were carried out at room temperature in a three-electrode cell using a CHI700E workstation (CH Instruments, Inc.). An aqueous solution of N_2_-saturated 0.5 M H_2_SO_4_ (pH = 0.3) was used as the electrolyte. The Pt thin film of the Pt/Si nanodiode electrode, an Ag/AgCl electrode (1 M KCl solution), and a Pt wire were used as the working electrode, reference electrode, and counter electrode, respectively. The potentials measured vs. Ag/AgCl were converted to the RHE scale according to

$${E}_{{\rm{RHE}}}={E}_{{\rm{Ag}}/{\rm{AgCl}}}+0.059{\rm{pH}}+{E}_{{\rm{Ag}}/{\rm{AgCl}}}^{0}={E}_{{\rm{Ag}}/{\rm{AgCl}}}+0.215,$$

where *E*_RHE_ is the potential after conversion to the RHE scale, and *E*_Ag/AgCl_ is the experimentally measured potential using a Ag/AgCl reference electrode; ${E}_{{\rm{Ag}}/{\rm{AgCl}}}^{0}$ = 0.1976 at 25 °C.

## Supplementary information

Supporting information

revised manuscript highlighted
