# Supplementary material for: Hot electron-driven electrocatalytic hydrogen evolution reaction on metal–semiconductor nanodiode electrodes
Source: Sci Rep. 2019 Apr 17;9:6208. doi: 10.1038/s41598-019-42566-3 (PMC6470139; doi:10.1038/s41598-019-42566-3)
Supplement: Supplementary file 1 — Supporting information [file 41598_2019_42566_MOESM1_ESM.pdf]

Supporting Information

Hot electron-driven electrocatalytic hydrogen evolution reaction on metal–semiconductor nanodiode electrodes

Ievgen I. Nedrygailov*[1], *Song Yi Moon*[1,2], *and Jeong Young Park*[1,2]*

[1] Center for Nanomaterials and Chemical Reactions, Institute for Basic Science, Daejeon 305-701, Republic of Korea

[2] Department of Chemistry and Graduate School of EEWS, Korea Advanced Institute of Science and Technology (KAIST) Daejeon 305-701, Republic of Korea

*To whom correspondence should be addressed. E-mail: jeongypark@kaist.ac.kr

KEYWORDS: electron transfer, electrochemistry, hot electron, hydrogen evolution, Schottky diode

1. Characterization of nanodiode electrodes.

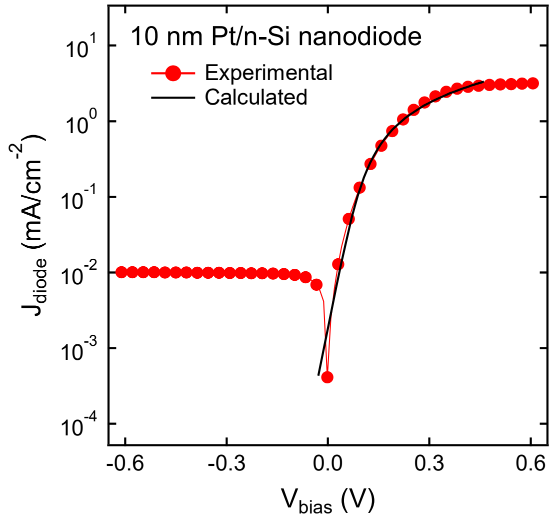

Figure S1. Typical current–voltage curves measured from the Pt/Si nanodiode electrodes. The solid black line shows the fit to the thermionic emission equation.

2. Determination of the onset potential for the hydrogen evolution reaction (HER).

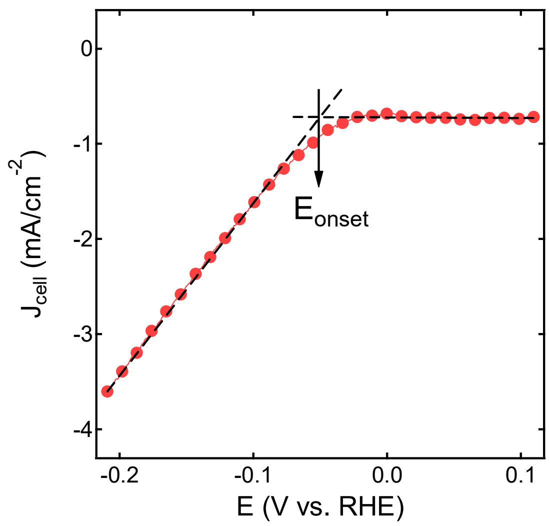

Figure S2. A typical linear sweep voltammetry (LSV) curve of the Pt/Si nanodiode electrode. The onset potential (E_{onset}) is determined as the potential where the tangents cross at the non-faradaic (horizontal line) and faradaic zones of the LSV curve.

3. Electrochemical impedance spectroscopy measurements. The electrochemical impedance spectroscopy measurements were performed using a VersaSTAT MC Electrochemical System (Princeton Applied Research). The data were collected at the potential of the working electrode E = 0.8 (V vs. RHE) at frequencies of f = 50 kHz – 10 Hz. Fitting of the experimental EIS data was performed using EC lab software. The semicircular portion of the Nyquist plot can be fitted using the R(RQ) equivalent circuit. Both the equivalent circuit and the fitting parameters are given in Supplementary Table S1. The artifact observed on the EIS spectrum at high frequencies is probably caused by significant impedance between the reference electrode and the test solution. A similar phenomenon was previously described elsewhere [D. Lloyd et al., Electrochim. Acta **109**, 843-851 (2013)].

**Fitting parameters**		**Equivalent circuit**
R_1	110.8 Ohm	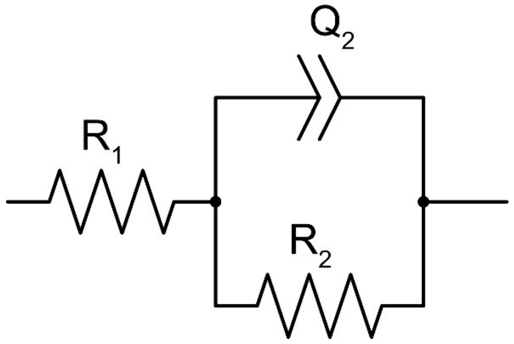
R_2	26.6 Ohm	
Q_2	0.187×10^{-3} F.s^(a_2-1)	
a_2	0.667	

Table S1. The equivalent circuit and fitting parameters for the 10 nm Pt/Si nanodiode electrode.

4. Characterization of Pt films and Pt/Si contacts.

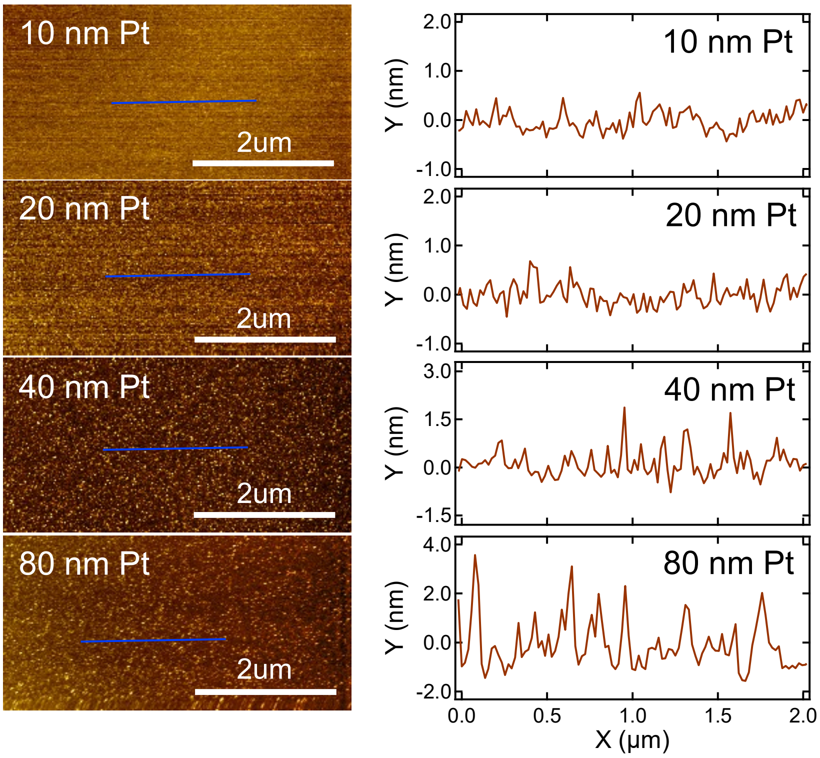

Figure S3. Atomic force microscopy (AFM) images of the surface of the Pt films with varied thickness.
